# A Case of Angular Curvature of Spine, Preceded by Lumbar Abscess, Treated by Leather Brace, Etc.*Read before Buffalo Medical Club, January 19, 1881.

**Published:** 1881-02

**Authors:** Bernard Bartow


### A CASE OF ANGULAR CURVATURE OF SPINE, PRECEDED BY LUMBAR ABSCESS, TREATED BY LEATHER BRACE, ETC.*

REPORTED BY BERNARD BARTOW, M. D.

Dec. 1st, 1880. Was consulted by Mr. B., for a tumor in the right lumbar region. The tumor was the size of a large orange, fluctuating, and but slightly painful upon pressure. Over the third and fourth lumbar vertebræ there was thickening and in-

* Read before Buffalo Medical Club, January 19, 1881.

duration of the overlying tissues, and slight pain when firm pressure was made at that point. No departure from the normal line of the spinal column was noticeable; the only symptoms pointing to the spine as the origin of the tumor being a peculiar straddling walk, and rigidity of the spine from muscular fixation, and the tenderness over the vertebræ before mentioned. Patient is a man of robust health and very active for his age; had been uniformly well until the appearance of this tumor. He stated that a year ago, by unexpectedly stepping off a high curbstone, his spine received a severe shock; the lameness following this jarring did not disappear for several months, it was confined principally to his spine and the muscles of his back. In July last he felt slight pains over the region of the kidneys, and shortly afterward felt a "lump" in the present site of the tumor. This continued to increase gradually and painlessly until of the size when first seen by myself. A good deal of discomfort from chafing of the part, and a considerable loss of strength and endurance were the principal inconveniences attending it.

The appearances were those of chronic or so-called "cold" abscess, and pointed to a probable spinal origin. I decided to empty the tumor of its contents, taking the precaution to preserve strict antiseptic conditions while doing so, and in the subsequent treatment of the affair. With the assistance of Dr. H. R. Hopkins this plan was carried out. The contents of the tumor consisted of blood pus, and broken down tissues, and amounted to about twelve fluid-ounces. The opening was a mere puncture sufficient to introduce a small drainage tube; the whole was covered with absorbent carbolized cotton and rubber tissue and properly secured. At the end of 48 hours the dressing was changed antiseptically; there had been about a half fluid ounce of discharge through the tube in the interval, while the wall of abscess was flat, and in the best condition to have the sac heal. Every 48 hours the dressing was renewed, the discharge rapidly diminishing, until a few drops only would ooze from the tube in the interval. At the end of three weeks the tube escaped from the opening, which promptly healed, and the whole matter appeared to be at an end.

Following the evacuation of the abscess the patient experienced no fever, no exhaustive suppuration, nor any of the formidable sequelae that have caused surgeons to advise against interfering with these abscesses that appear to be connected with spinal caries. On the contrary, in this instance, there was great relief from general discomfort and anxiety, while the patient's appetite and strength perceptibly improved. A few days after the opening had closed a small amount of fluid was observed to have re-formed near the former site of the abscess, being this time nearer to the spinal column. This was opened also in a manner similar to the first, discharging a fluid ounce of pus; a drainage tube was again inserted and remains in at the present time. As before, no constitutional disturbance followed, and at no time, during the ensuing four weeks, did the amount of discharge during the interval of dressing exceed one fluid dram.

The tenderness over the third and fourth vertebræ was continuous from the beginning, and during these latter weeks increased considerably; coincident with this increase of pain there was observed a slight posterior bulging of the third and fourth lumbar vertebræ. Owing to the low position of the disease and the great superincumbent weight, this curvature increased very rapidly, and in three weeks was a distinctly defined angular curve involving those two bones.

The rigidity of the spine and the straddling gait, before mentioned, became more noticeable, while all efforts by which the spine was brought into action were made with considerable difficulty. The demand for mechanical support becoming very apparent, the plaster of Paris "jacket" was applied Jan. 7th. This was removed as soon as it had become hard and used as a mould for a plaster cast of the patient's body; upon this casting was formed a brace made by wrapping the form with a piece of belting leather $\frac{3}{16}$ inch in thickness, and with a strong cord closely winding or "serving" the leather to the form in its entire length. After drying, the leather was trimmed, holes punched quite closely over its entire surface, and the whole secured upon the patient by straps and buckles in front. It was a duplicate in form of the "jacket" plaster, but much stronger, lighter, and in many respects, more comfortable and capable of being removed when desirable.*

When applied, the brace afforded requisite support to the spine, as was seen in the greater freedom with which the patient could walk, lie down, rise up, turn over, etc., showing the spine to have been placed wholly at rest. Allowance was made in the leather brace to admit of abscess dressing, the removable quality in the brace excluding the necessity of an opening in it, for this special purpose. The tenderness over the vertebræ has greatly subsided, and the extension and support of the spine has materially lessened the prominence of the angle; the discharge from the abscess does not exceed a fluid dram in 48 hours. Everything indicates the conditions for consolidation of the diseased vertebræ to be quite perfect, and the prognosis to possess but little of an unfavorable character.

The points of interest illustrated by this case are the antiseptic treatment of the abscess, and the substitution of a removable leather brace for the plaster of Paris jacket.

Of the first, it may be said, that the dangerous consequences attending the opening of this form of abscess in the manner usually employed, have not been exaggerated by surgical writers, and particularly is this the case when occurring in a person of advanced years. When, for any reason, it has been considered necessary to evacuate such an abscess, it has been advised to do so with care as to the exclusion of air from the sac—the exhaustive suppuration and rapid increase in the associated spinal disease often ensuing upon the non-observance of such care.

If analogous conditions, in which the employment of Lister's method of antisepsis has operated so successfully, have been slow in appealing to the *unreason* of some surgeons, will not

* This process of moulding leather upon plaster forms was described in the BUFFALO MEDICAL AND SURGICAL JOURNAL, Oct., 1880.

cases like the one in point carry a little convincing evidence to them? Holmes* mentions having employed Lister's method in the opening of spinal abscesses, with great advantage to their subsequent progress. Sayre unhesitatingly advises the opening of such abscesses under antiseptic conditions. Such authority in itself ought to make surgeons more truly conservative in their opinions, and particularly in their practice, when this idea is involved.

The case in point is unique in presenting all the features to distinguish it as an unmistakable disease, in which the attending dangers have been avoided by this practice. Under any other conditions I should not have dared to interfere by opening the abscess, and I should have looked forward even with great apprehension to the spontaneous evacuation of the abscess. Regarding the use of a leather brace for spinal support, I will only say that thus far I have employed it in six cases—three of angular, and three of lateral curvature, and find the support derived from it as great as I have observed from the plaster Paris "jacket," while in addition many of the disagreeable and objectionable qualities of the latter are avoided without a proportionately great increase of the labor and expense involved.

* Surgical Diseases of Childhood.

